# Increased expression of TBC1D10B as a potential prognostic and immunotherapy relevant biomarker in liver hepatocellular carcinoma

**DOI:** 10.1038/s41598-022-20341-1

**Published:** 2023-01-07

**Authors:** Li Fan, Yongmei Tang, Jingjing Li, Wenjie Huang

**Affiliations:** Department of Reproductive Medicine, Liuzhou Maternity and Child Healthcare Hospital, Liuzhou, 545001 Guangxi China

**Keywords:** Cancer, Computational biology and bioinformatics

## Abstract

The TBC1 domain family member 10B (EPI64B/TBC1D10B), a member of the RabGAP EPI64 subfamily, contains a TBC domain that confers GTPase-activating protein activity. Even though overexpression of TBC1D10B has been reported to promote tumor invasion and metastasis in gastric adenocarcinoma, the prognostic value of TBC1D10B and its correlation with DNA methylation and immune infiltration in hepatocellular carcinoma are still not known. Transcriptional expression profiles of TBC1D10B between hepatocellular carcinoma tissues and normal tissues were downloaded from The Cancer Genome Atlas and Gene Expression Omnibus. The Clinical Proteomic Tumor Analysis Consortium and the Human Protein Atlas were used to assess the TBC1D10B protein expression. The biological functions of TBC1D10B were evaluated by the Metascape database and by Gene Set Enrichment Analysis (GSEA). Receiver operating characteristic (ROC) curve analysis was used to distinguish hepatocellular carcinoma from adjacent normal tissues. The effect of TBC1D10B on survival was estimated using the Kaplan–Meier method. DNA methylation in the TBC1D10B gene was assessed using the online MEXPRESS and MethSurv tools. The association between TBC1D10B mRNA expression and immune cell infiltration was investigated by the TIMER2 web server, tumor immune estimation resource and single-sample GSEA. This study found that TBC1D10B is highly expressed in hepatocellular carcinoma and that increased TBC1D10B mRNA expression is associated with female sex, lower Body Mass Index, high level of alpha fetal protein, and worse clinical stages. The mRNA and protein levels of TBC1D10B were verified in cells. Functional annotation indicated enrichment with negative regulation of the cell cycle, extracellular matrix, and corresponding pathways in the high-TBC1D10B phenotype. The ROC curve analysis showed that, with a cutoff level of 2.912, the accuracy, sensitive, and specificity in differentiate TBC1D10B hepatocellular carcinoma from adjacent controls were 0.931, 0.920, and 0.802, respectively. Kaplan–Meier survival analysis showed that hepatocellular carcinoma patients with high TBC1D10B had a worse prognosis than those with low TBC1D10B, especially in patients with a weight below 70 kg, height above 170 cm, and histological G2 and G3. We also found that the methylation of TBC1D10B was associated with the prognosis in patients with hepatocellular carcinoma. Moreover, correlation analysis indicated that TBC1D10B mRNA expression was positively correlated with infiltration levels of most immune cells, but negatively correlated with Th17 and cytotoxic cells infiltration. Our study indicates that increased TBC1D10B expression in hepatocellular carcinoma may play a role in tumorigenesis by regulating the cell cycle and extracellular matrix. TBC1D10B may be a novel prognostic and predictive marker and immune therapeutic target in hepatocellular carcinoma patients.

## Introduction

Hepatocellular carcinoma, the third leading cause of cancer death, is the most frequent primary liver malignancy and an important medical problem. Most hepatocellular carcinoma cases occur in eastern Asia and sub-Saharan Africa, where the most prevalent risk factors are aflatoxin B1 and hepatitis B exposure^1^. The development of hepatocellular carcinoma is a multiple process that involves sustained inflammatory conditions, including hepatocyte necrosis and regeneration, related to fibrotic deposition^2^. Although several studies have shown that surgical resection, ablation, transplantation, chemoembolization^3^, and the tyrosine-kinase inhibitors lenvatinib^4^ and sorafenib^5^ are treatments with survival benefits, the characteristics of multifocal development and drug-resistance preclude treatment being curative in most hepatocellular carcinoma cases^6,7^. Given that hepatocellular carcinoma is the result of epigenetic modifications and somatic genomic alterations in passenger and driver genes^2^, the further understanding of the molecular classifications of hepatocellular carcinoma that predict disease progression or recurrence is of paramount importance.

The Rab GTPase family comprises a large number of small GTPases that exist in two conformational states: an inactive GDP-bound protein and an active GTP-bound protein^8,9^. Rab GTPase is involved in regulating intracellular vesicular transport spatially and temporally by switching between inactive and active states^10^, which are catalyzed by GTPase activating proteins (GAPs) and guanine nucleotide exchange factors (GEFs), respectively^11^. More importantly, Rabs and their effectors are found to be overexpression or aberrant function in hepatocellular carcinoma, including Rab1A^12^, Rab5A^13^, Rab18^14^, Rab31^15^ and Rab27A^16^. The Tre-2/Bub2/Cdc16 (TBC) domain is a conserved catalytic domain with ~ 200 amino acids presenting in various molecules in eukaryotic organisms^17^ as Rab-GAPs^18^. The TBC1D10 subfamily members (TBC1D10A/EPI64, TB1D10B/EPI64B and TBC1D10C/EPI64C/carabin) possess a TBC domain and have been reported to exhibit GAP activities toward all Ras and certain Rabs^19,20^. Each TBC1D10 member exhibits distinct Ras/Rab specificities, as well as distinct biological functions: TBC1D10A exhibits its effects on angiogenesis^21^ and selective autophagy^22^ through binding to Rab3A and 35, respectively; TBC1D10B participate in regulating vesicle-mediated membrane traffic by binding to Rab35, 3A, and 27A^23–25^; and TBC1D10C serves as a negative modulator in T-cell activation through dual inhibition of both calcineurin and Ras pathways^26^. Additionally, the overexpression of EPI64 has been proven to contribute to exosome secretion in lung cancer^17^, and aberrant activation of the Ras pathways has been causally observed in a human hepatocellular carcinoma that is characterized by shorter survival^27^, indicating potential roles of TBC1D10 in tumor progression and prognosis. In light of the fact that the knockdown of TBC1D10B attenuates the viability, apoptosis, and proliferation ability of gastric adenocarcinoma cells^28^, we asked whether increased TBC1D10B in hepatocellular carcinoma may play a role in tumorigenesis and have prognostic value for clinical outcomes.

Therefore, we carried out a bioinformatics analysis to identify the significance of TBC1D10B in liver hepatocellular carcinoma (LIHC) tumorigenesis and prognosis. We found an overexpression of TBC1D10B in LIHC and explored its potential role in LIHC tumorigenesis. Then, a significant correlation was observed between TBC1D10B and several clinicopathological characteristics. Furthermore, we identified the diagnostic and prognostic values of TBC1D10B. Our study provides a novel insight into the underlying mechanisms of LIHC tumorigenesis and reveals TBC1D10B as a potential diagnostic and prognostic biomarker in LIHC.

## Materials and methods

### Gene differential expression analysis

RNA sequencing and related clinical data were downloaded from the Cancer Genome Atlas (TCGA) ^33typesofcancer^ using UCSC Xena platform (http://xena.ucsc.edu/), an online tool for the exploration of gene expression and clinical phenotype data. Expression data were Log2 transformed, and Wilcoxon rank sum test conducted on these tumor types; *P* < 0.05 were considered to indicate differential expression between tumor and normal tissues. The data analysis was conducted using R software (version 3.6.3; https://www.R-project.org), and the R package “*ggplot2*” was used to draw box plots. Then, we downloaded high-throughput RNA-sequencing data of 424 samples from the TCGA data set. The 424 samples included 50 matched normal-liver cancer samples (normal samples adjacent to tumors for a total of 100 samples) and 324 unmatched liver cancers. The expression of TBC1D10B was evaluated using the downloaded data, and expression levels were compared between 50 cancer samples and 50 matched adjacent normal samples in LIHC.

In total, 374 LIHC patients were separated into high- and low-TBC1D10B expression groups based on the median TBC1D10B value. Differentially expressed gene (DEG) analyses between the two groups by a two-tailed hypothetical test were conducted using the R package “*DESeq2*”^29^, based on the negative binomial generated linear models in which the log-fold change is larger than 1.5 and the adjusted *P*-value less than 0.05. Visualizations of DEGs, such as heatmaps and volcano plots, were generated using the R package “*pheatmap*” and “*EnhancedVolcano*.” As the TCGA cohort has already obtained Ethics Committee Approval, this study did not require additional approval.

In addition, we selected 4 datasets: GSE45267 (GLP570), GSE101685 (GLP570), GSE87630 (GPL6947) and GSE57957 (GPL10558) from the Gene Expression Omnibus (GEO, https://www.ncbi.nlm.nih.gov/geo/) for external verification of TBC1D1B expression in LIHC. GSE45267 obtained 48 primary HCC samples and 39 non-cancerous tissues, whereas GSE101685 included 8 normal tissue and 24 HCC patients with T1, T3a, and T3b. For GSE87630, there were 64 tumor tissue samples and 30 non-tumor tissue samples, whereas GSE57957 included 39 primary HCC tissue samples and paired normal tissue samples.

### Protein expression studies and immunohistochemistry (IHC) staining

To evaluate the difference in TBC1D10B protein expression levels between a primary tumor and normal tissue, we implemented UALCAN (http://ualcan.path.uab.edu/analysis-prot.html) to present a thorough analysis of TBC1D10B protein expression and its phosphorylation from the Clinical Proteomic Tumor Analysis Consortium data set. Meanwhile, the IHC images of TBC1D10B protein expression in normal tissue and liver LIHC tissue were downloaded from the Human Protein Atlas (HPA) (http://www.proteinatlas.org/) and analyzed.

### Cell culture, RNA extraction, and quantitative real-time PCR (qRT-PCR)

A total of four cell lines including human hepatoma cell lines (Huh-7, Bel7402 and Sk-hep-1) and the WRL68 non-tumor human embryo liver cells, which were obtained from Cell Bank of Chinese Academy of Science (Shanghai, China). Huh-7, BEL-7402 and SK-HEP-1 were cultured in DMEM (Gibco, Carlsbad, CA, USA) with 10% fetal bovine serum (Gibco, Carlsbad, CA, USA). WRL68 was cultured in RPMI-1640 (Gibco, Carlsbad, CA, USA) containing 10% fetal bovine serum. All cells were maintained in a humidified incubator with 37 ℃, 5% CO_2_. qRT-PCR was performed following the manufacturer’s instruction. The total RNA was extracted by Trizol (Sigma-Aldrich). The reverse transcriptase reaction was performed using RevertAid RT Kit (Thermo Scientific). The PCR reaction was performed using SYBR Green (TOYOBO QPK-201, Tokyo, Japan). Data were analyzed using the 2^−ΔΔCt^ method. The primers sequences for TBC1D10B and GAPDH are as follows: TBC1D10B forward primer (5′-CAGGAGGCATTGCTGATGAT-3′) and TBC1D10B reverse primer (5′-GAAGGCTGGGGCTCATTT-3′); GAPDH forward primer (5′-CCATCCCTTCCCTCACCCACTG-3′) and GAPDH reverse primer (5′-TCTCCCGCTCCTTCTCCTGTTTC-3′).

### Western blot analysis

Western blot was performed as previously described^30^. Briefly, total protein was extracted from cells by using RIPA extraction buffer with protease inhibitors (1:100, BestBio Science, Shanghai, China) according to the manufacturer’s instructions. After electrophoresis, proteins were electroeluted onto a polyvinylidenedifluoride (PVDF) memberane (Invitrogen). The membrane was blocked with Tris-buffered saline containing 0.1% Tween 20 and 5% milk for 2 h at room temperature, and then incubated with the following antibodies against TBC1D10B (abs103211, Absin, China), β-actin (81115-1-RR, Proteintech, China) at 4℃ overnight. The membrane was incubated at room temperature for 1 h with HRP-conjugated secondary antibodies. Proteins were visualized by chemiluminescence.

### Functional annotation of TBC1D10B-associated DEGs in LIHC

Functional annotation of the identified DEGs was performed using the Metascape database and online tool^31^. The enrichment factors were larger than 1.5, and the minimum counts larger than 3. For the analysis, the *P*-value threshold was set to 0.01. The Gene Set Enrichment Analysis (GSEA) of DEGs between the two groups was performed using the R package “*clusterProfiler*”^32^. The gene set collection C2 (curated gene sets) was obtained from the Molecular Signature Database as the reference gene sets. We finally identified a total of 772 clusters for which a false discovery rate (FDR) of < 0.25 and a P-value of < 0.05 were considered significant. Protein–protein interaction networks were constructed based on the STRING database (https://string-db.org/) and were visualized in Cytoscape software^33^.

### Correlation analysis for TBC1D10B expression and the clinicopathological characteristics of LIHC

The Wilcoxon rank sum test (continuous variables) or Pearson’s chi-square test (rank variables) was used to examine the relationship between TBC1D10B expression levels and clinicopathological characteristics. Visualizations of clinical phenotype correlations were generated using the R package “*ggplot2*.”

### Clinical significance of TBC1D10B expression in LIHC

Receiver operating characteristic (ROC) analysis was used to estimate the predictive value of TBC1D10B for LIHC diagnoses. Information on LIHC patients’ clinical outcomes, including overall survival, disease-specific survival, and progression-free interval, was obtained from a published study^34^. The R packages “*survminer*” and “survival” were applied to Kaplan–Meier (K-M) analysis and the clinicopathological subgroup study. All statistical analyses were carried out in R v. 3.6.3. with a *P*-value of ≤ 0.05.

### Analysis of TBC1D10B methylation and prognosis

The methylation data of TBC1D10B from multiple probes (e.g., cg05099952, cg26843872) were obtained from the MEXPRESS website (https://mexpress.be/). Next, we analyzed the prognostic value of the TBC1D10B methylation level in LIHC using MethSurv (https://biit.cs.ut.ee/methsurv/), a web tool for providing survival analysis based on TCGA data.

### Association of TBC1D10B expression and immune cell infiltration in LIHC tumors

We used the “Immune-Gene” module of TMIER.2 to explore the correlation between TBC1D10B expression and immune infiltrates (B cells, CD4^+^ T cells, CD8^+^ cells, macrophages, neutrophils, and dendritic cells). In addition, we performed GSEA using the package “*GSVA*” to present the infiltration enrichment of 19 common immune cells, including activated DCs (aDCs), immature DCs (iDCs), plasmacytoid DCs, T cells, T helper (Th) cells, type 1 Th cells (Th1s), type 2 Th cells (Th2s), type 17 Th cells (Th17s), T gamma delta cells, T effector memory (Tem) cells, regulatory T (Treg) cells, T central memory cells, T follicular helper (Tfh) cells, eosinophils, cytotoxic cells, mast cells, natural killer (NK) cells, NK 56− cells, and NK 56+ cells. Then, the relationship between TBC1D10B and immune infiltration was estimated by Spearman’s analysis and the Wilcoxon rank sum test.

### Ethical approval

All data analyzed in the present study are available in the TCGA database. No ethics approval was required for the purpose of this study.

## Results

### Expression profiles of TBC1D10B in pan-cancer and related DEGs in LIHC

**Figure 1:**
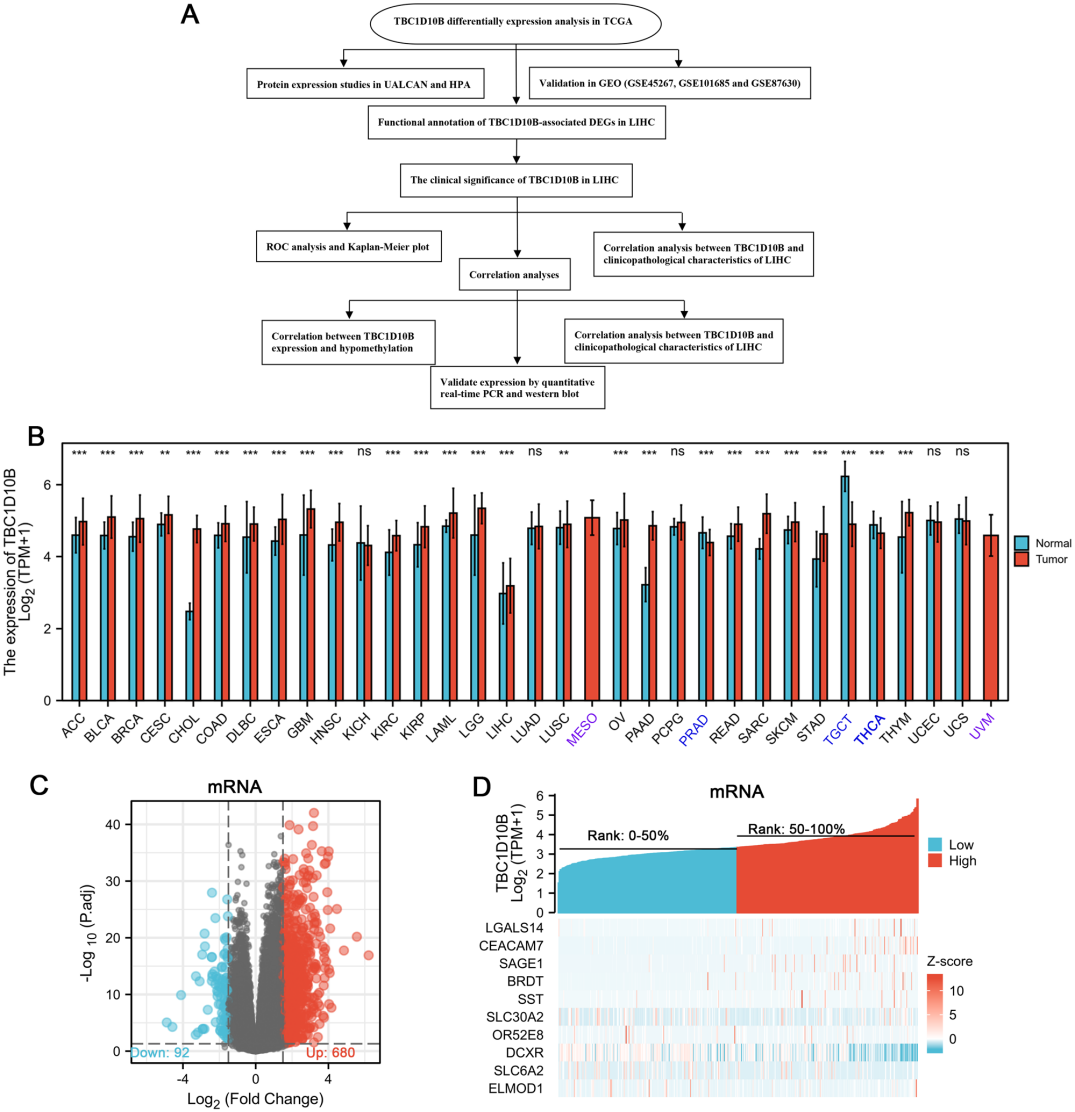
Differential mRNA expression profiles in liver hepatocellular carcinoma (LIHC) patients. (**A**) Flow chart of the study. (**B)** Expression level of TBC1D10C gene in 33 types of tumors. Volcano plots (**C**) and a heat map (**D**) depicting the mRNA expression profiles are shown. Differentially expressed genes that are up- or downregulated at least 1.5-fold. ns, *P* > 0.05; **P < 0.01; ***P < 0.001.

To describe our study more clearly, a flow chart was developed to systematically describe our study (Fig. 1A). We compared TBC1D10B mRNA expression levels in different cancers based on TCGA data. As shown in Fig. 1B, TBC1D10B was overexpressed in 23 of 33 cancer types. 374 patients were divided into low- and high-TBC1D10B expression groups based on the median TBC1D10B expression in LIHC tumors. Comparing the expression levels between the two groups, we found that 772 mRNAs (680 upregulated and 92 downregulated; Fig. 1C), 1 miRNA (1 upregulated; Supplementary Fig. 1A), and 388 lncRNAs (322 upregulated and 66 downregulated; Supplementary Fig. 1B) were defined as DEGs (*P* < 0.05; absolute value of fold change > 1.5) in the high-TBC1D10B group. Representative DEGs were also illustrated by heatmaps (Fig. 1D and Supplementary Fig. 1C,D).

### The mRNA and protein expression of TBC1D10B in LIHC

**Figure 2:**
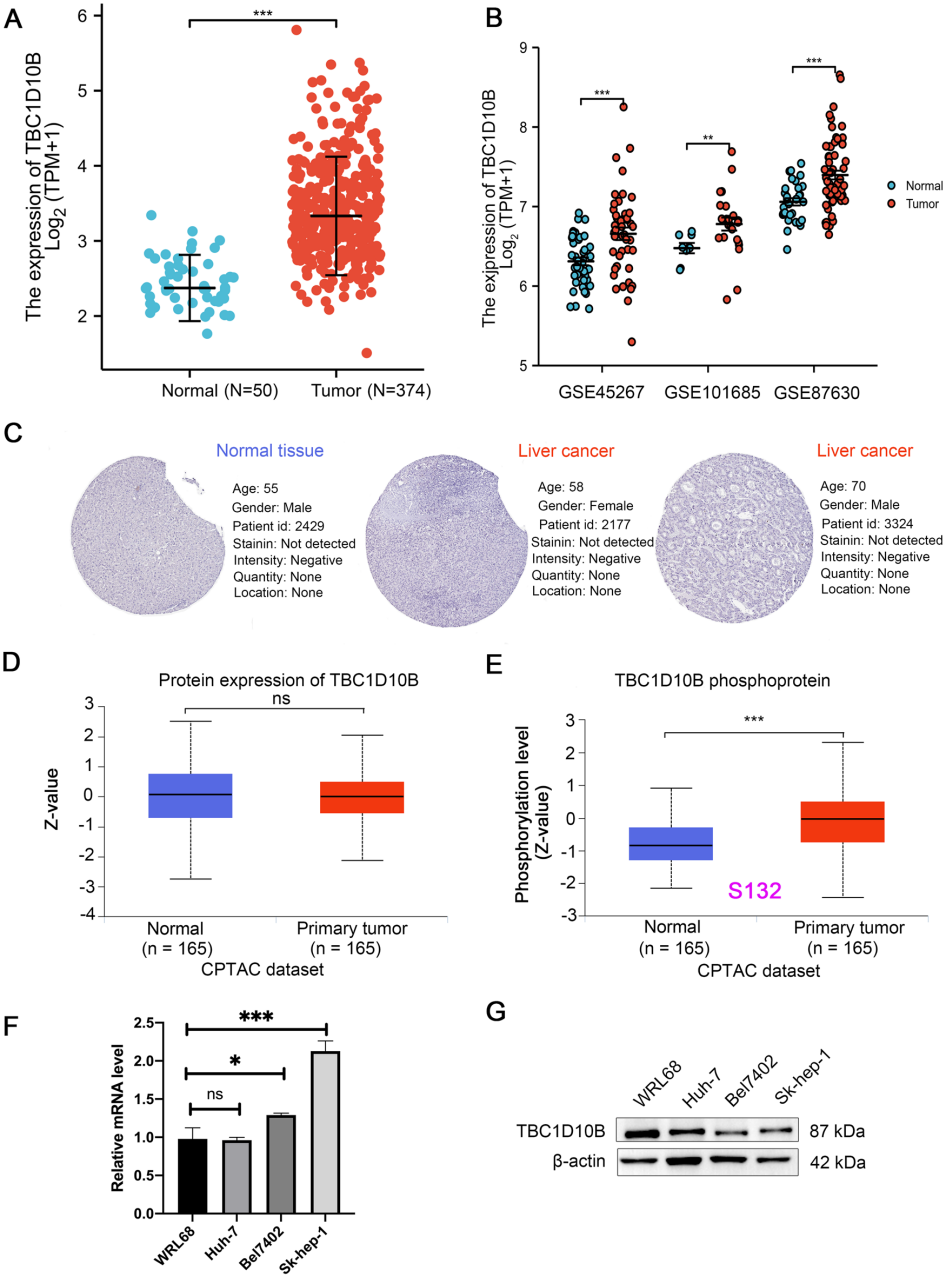
The mRNA and protein expression of TBC1D10B in LIHC. (**A**) Expression of TBC1D10B mRNA in 50 LIHC and 374 adjacent normal samples. (**B**) The mRNA expression of TBC1D10B in GEO database (GSE45267, GSE101685 and GSE87630). (**C,D**) The protein expression levels of TBC1D10B based on CPTAC and HPA database. (**E**) The expression level of TBC1D10B phosphoprotein (NP_056342.3, S132 site) between primary liver cancer tissue and normal tissue was analyzed via UALCAN. (**F,G**) mRNA levels (**F**) and protein levels (**G**) of TBC1D10B in human hepatoma cell lines (Huh-7, Bel7402 and Sk-hep-1) and non-tumor human embryo liver cell (WRL68) were determined by q-RT-PCR and Western blotting. *P < 0.05; ***P < 0.001.

To further determine the mRNA and protein expression of TBC1D10B in LIHC, we used data from TCGA and HPA to perform the analysis. As shown in Supplementary Fig. 2A,B, a paired data analysis of tumor and matched nearby tissue from TCGA and GEO showed increased TBC1D10B mRNA in LIHC. Unpaired data analysis also showed that the high mRNA expression levels of TBC1D10B in LIHC tissues compared to those in adjacent tissues (Fig. 2A,B). However, the observed changes in TBC1D10B protein in LIHC were not due to parallel changes in TBC1D10B RNA levels, and no TBC1D10B expression was detected by immunohistochemistry in normal and tumor tissues (Fig. 2C,D). Interestingly, while the TBC1D10B total protein levels remained unchanged, the phosphorylated TBC1D10B level at S132 was increased (Fig. 2E). In addition, this was validated at both the mRNA and protein levels in liver cancer cells and normal liver cells (Fig. 2F,G). This finding merits further molecular assays for further exploration of the potential role of TBC1D10B phosphorylation of S132 in tumorigenesis.

### Functional annotation of TBC1D10B-association DEGs in LIHC

**Figure 3:**
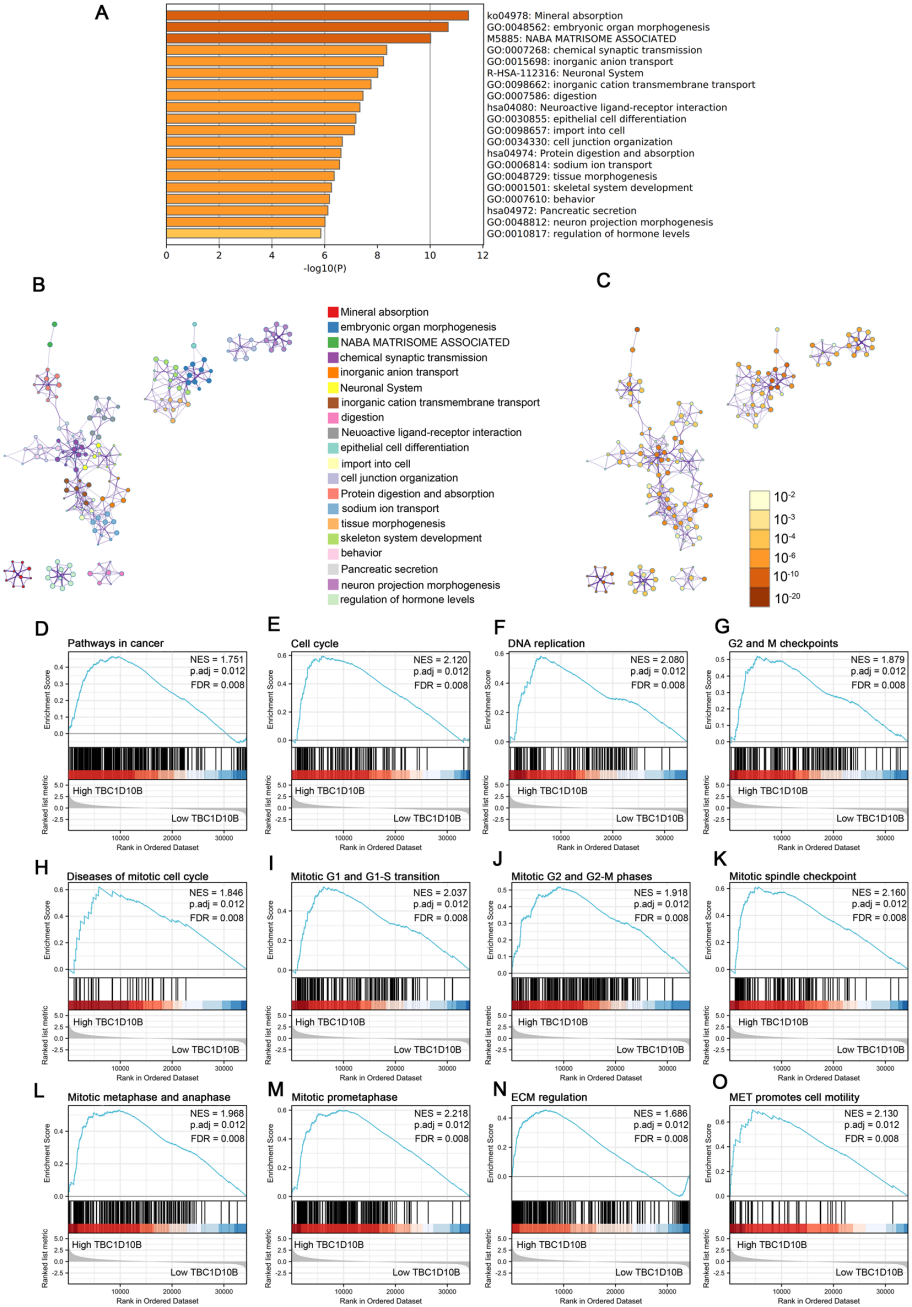
Functional annotation differential expressed genes (DEGs) in LIHC patients with different expression levels of TBC1D10B. We used the Metascape database to perform the functional annotation based on 772 differentially expressed mRNAs between high- and low-TBC1D10B expression groups. Based on the kappa threshold of 0.3, all statistically enriched terms were identified and then hierarchically in to a tree (**A**). The network layout was generated using representative terms from the cluster (**B**). Node size correlates with the number of input genes, different colors represent different cluster. The thickness of the edge based on the similarity score. The same enrichment network presents nodes colored by the P-value (**C**). Representative Gene Set Enrichment Analysis of differentially expressed mRNA between the high TBC1D10B expression group and the low expression group (**D–O**).

We used the software Metascape to evaluate the function of TBC1D10B-associated DEGs in LIHC. As presented in Fig. 3A–C and Table 1, some LIHC-related pathways were enriched, including epithelial cell differentiation (GO: 0030855), cell junction organization (GO: 0034330), skeletal system development (GO: 0001501) and chemical synaptic transmission (GO: 0007268). Similarly, GSEA showed that TBC1D10B-associated DEGs significantly enriched in cell proliferation-related clusters (Fig. 3E–M), such as diseases of the mitotic cell cycle (normalized enrichment score [NES] = 1.846; adjust *P* = 0.012; FDR = 0.008), mitotic spindle checkpoint (NES = 2.160; adjust *P* = 0.012; FDR = 0.008), mitotic metaphase and anaphase (NES = 1.968; adjust *P* = 0.012; FDR = 0.008), mitotic G1 and G1-S transition (NES = 2.037; adjust *P* = 0.012; FDR = 0.008), and mitotic prometaphase (NES = 2.218; adjust *P* = 0.012; FDR = 0.008). TBC1D10B-associated DEGs were also enriched in cancer pathways (Fig. 3D). More importantly, TBC1D10B-associated DEGs were associated with extracellular matrix (ECM) regulation and the MET gene (Fig. 3N–O), which were usually involved in both development and cancer progression.

**Table 1:** Top 20 clusters in pathway and process enrichment analysis of DEGs in LIHC patients with distinct TBC1D1B levels.

Cluster	Description	P value	Enrichment factor	FDR
ko04978	Mineral absorption	< 0.001	1.97	< 0.001
GO:0048562	Embryonic organ morphogenesis	< 0.001	4.19	< 0.001
M5885	Naba Matrisome Associated	< 0.001	7.08	< 0.001
GO:0007268	Chemical synaptic transmission	< 0.001	6.42	< 0.001
GO:0015698	Inorganic anion transport	< 0.001	2.88	< 0.001
R-HSA-112316	Neuronal system	< 0.001	4.46	< 0.001
GO:0098662	Inorganic cation transmembrane transport	< 0.001	6.68	< 0.001
GO:0007586	Digestion	< 0.001	2.36	< 0.001
hsa04080	Neuroactive ligand-receptor interaction	< 0.001	3.41	< 0.001
GO:0030855	Epithelial cell differentiation	< 0.001	5.64	< 0.001
GO:0098657	Import into cell	< 0.001	3.01	< 0.001
GO:0034330	Cell junction organization	< 0.001	5.77	< 0.001
hsa04974	Protein digestion and absorption	< 0.001	1.83	< 0.001
GO:0006814	Sodium ion transport	< 0.001	3.01	< 0.001
GO:0048729	Tissue morphogenesis	< 0.001	5.24	< 0.001
GO:0001501	Skeletal system development	< 0.001	4.59	< 0.001
GO:0007610	Behavior	< 0.001	4.98	< 0.001
hsa04972	Pancreatic secretion	< 0.001	1.83	< 0.001
GO:0048812	Neuron projection morphogenesis	< 0.001	5.11	< 0.001
GO:0010817	Regulation of hormone levels	< 0.001	4.46	< 0.001

*FDR* false discovery rate.

### Relationships between TBC1D10B mRNA levels and the clinical pathological characteristics of LIHC patients

**Figure 4:**
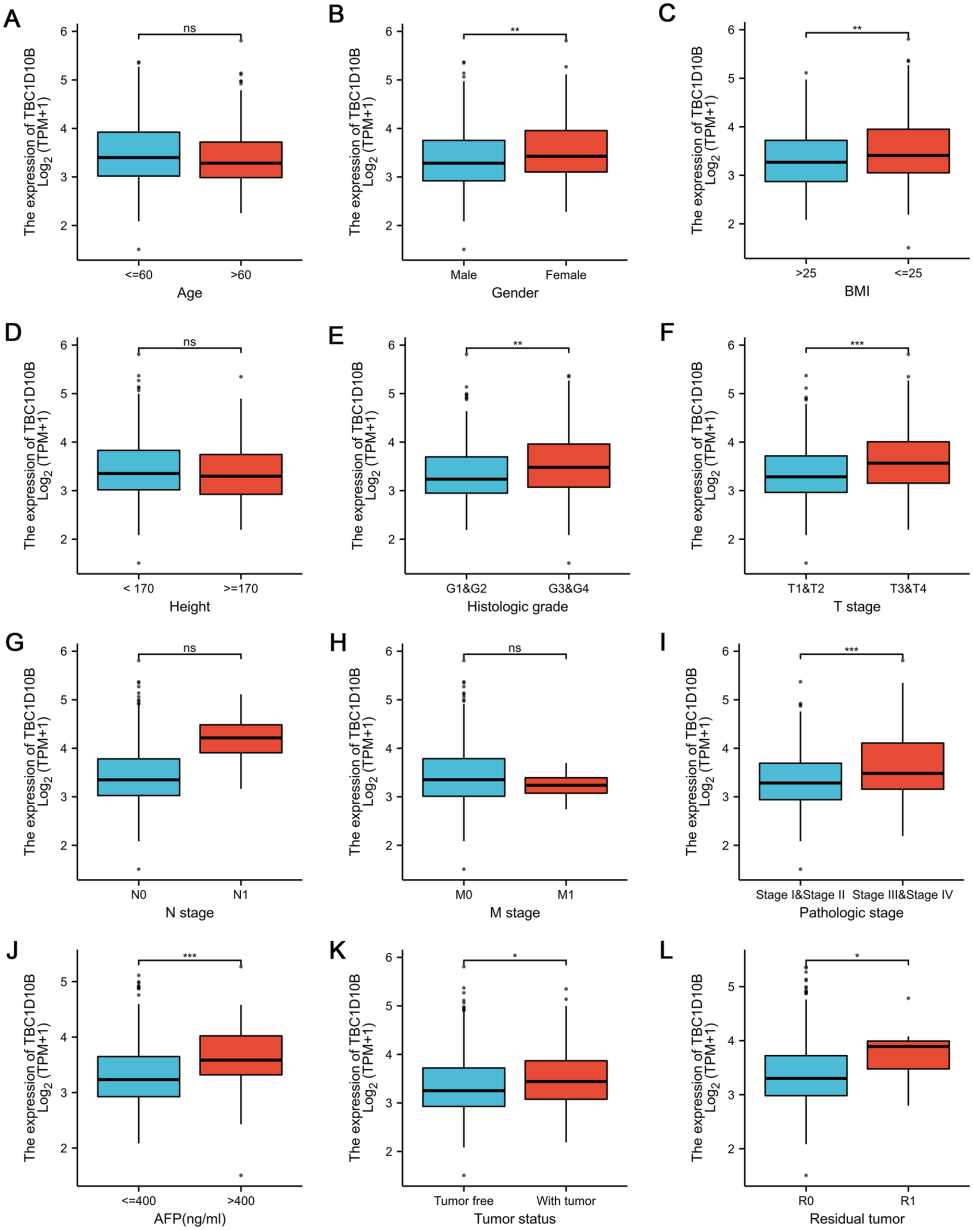
Association between TBC1D10B mRNA levels and clinicopathological features in LIHC. Based on the analysis of TCGA data, the expression levels of the TBC1D10B gene were positively correlated with gender was female (**A**), body mass index greater than or equal to 25 (**C**), high histologic grade (**E**), T stage (**F**), pathological stage (**I**), AFP level greater than 400 ng/mL (**J**), tumor status (**K**) and residual tumor (**L**), but not with age (**A**), height (**D**), N stage (**G**) and M stage (**H**). *ns* no significance, *P < 0.05, **P < 0.01, ***P < 0.001.

Next, we analyzed the relationship between TBC1D10B expression and clinical pathological characteristics in the TCGA data set, as shown in Table 2 and Fig. 4. High TBC1D10B expression was associated with female patients (Fig. 4B), non-overweight patients (body mass index [BMI] ≤ 25; Fig. 4C), alpha-fetoprotein elevation (AFP > 400; Fig. 4J), tumor bearing (Fig. 4K), residual tumor (R1; Fig. 4L), high histologic grade (grade 3/4; Fig. 4E), and more severe T stages (T3 and T4; Fig. 4F) and pathologic stages (stages III and IV; Fig. 4I). However, no statistically significant correlation was observed between high TBC1D10B expression and other clinical pathological characteristics, including age (Fig. 4A), height (Fig. 4D), T stage (Fig. 4G), and M stage (Fig. 4H). Therefore, elevated TBC1D10B may have at least partially acted as a biomarker of poor prognosis in LIHC.

**Table 2:** Clinicopathological characteristics of LIHC patients with differential TBC1D10B expression.

Characteristic	Total	Low expression of TBC1D10B (187)	High expression of TBC1D10B (187)	*p*-value
**T stage**				**0.003**
T1	183 (49.4%)	106 (28.6%)	77 (20.8%)	
T2	95 (25.6%)	47 (12.7%)	48 (12.9%)	
T3	80 (21.5%)	28 (7.5%)	52 (14%)	
T4	13 (3.5%)	4 (1.1%)	9 (2.4%)	
**N stage**				0.623
N0	154 (98.5%)	124 (48.1%)	130 (50.4%)	
N1	4 (1.6%)	1 (0.4%)	3 (1.2%)	
**M stage**				0.361
M0	268 (98.5%)	130 (47.8%)	138 (50.7%)	
M1	4 (1.5%)	3 (1.1%)	1 (0.4%)	
**Pathologic stage**				**0.001**
Stage I	173 (49.4%)	98 (28%)	75 (21.4%)	
Stage II	87 (24.9%)	44 (12.6%)	43 (12.3%)	
Stage III	85 (24.3%)	28 (8%)	57 (16.3%)	
Stage IV	5 (1.4%)	4 (1.1%)	1 (0.3%)	
**Tumor status**				**0.045**
Tumor free	202 (56.9%)	110 (31%)	92 (25.9%)	
With tumor	153 (43.1%)	66 (18.6%)	87 (24.5%)	
**Gender**				0.122
Female	121 (32.4%)	53 (14.2%)	68 (18.2%)	
Male	253 (67.6%)	134 (35.8%)	119 (31.8%)	
**Age**				0.055
≤ 60	177 (47.5%)	79 (21.2%)	98 (26.3%)	
> 60	196 (52.6%)	108 (29%)	88 (23.6%)	
**BMI**				0.091
≤ 25	77 (52.5%)	80 (23.7%)	97 (28.8%)	
> 25	160 (47.5%)	88 (26.1%)	72 (21.4%)	
**Residual tumor**				**0.025**
R0	327 (94.8%)	171 (49.6%)	156 (45.2%)	
R1	17 (5%)	4 (1.2%)	13 (3.8%)	
R2	1 (0.3%)	1 (0.3%)	0 (0%)	
**Histologic grade**				**0.006**
G1	55 (14.9%)	34 (9.2%)	21 (5.7%)	
G2	178 (48.3%)	98 (26.6%)	80 (21.7%)	
G3	124 (33.6%)	48 (13%)	76 (20.6%)	
G4	12 (3.3%)	4 (1.1%)	8 (2.2%)	
**AFP (ng/ml)**				** < 0.001**
≤ 400	215 (76.8%)	126 (45%)	89 (31.8%)	
> 400	65 (23.2%)	18 (6.4%)	47 (16.8%)	

Significant values are in [bold].

*LIHC* liver hepatocellular carcinoma, *AFP* alpha-fetoprotein, *BMI* body mass index.

### Predictive value of TBC1D10B for LIHC diagnosis and prognosis.

**Figure 5:**
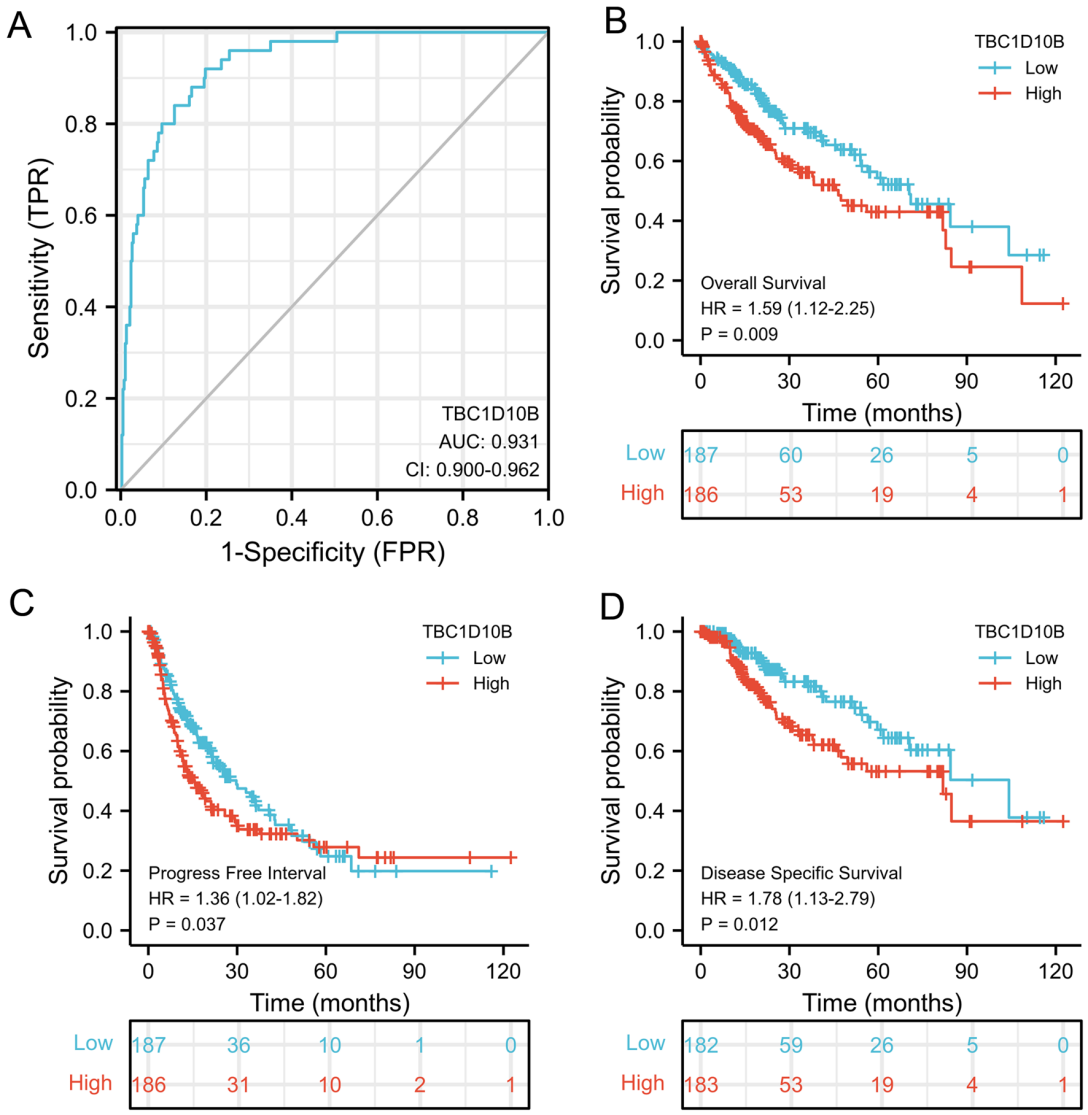
ROC and Kaplan–Meier curves for TBC1D10B gene in LIHC. (**A**) Receiver operating characteristic (ROC) curve for TBC1D10B that differentiates LIHC tissues from healthy controls. (TBC1D10B showed excellent performance as revealed by a ROC curve with an area under the ROC curve (AUC) of 0.93). (**B–D**) Kaplan–Meier analysis of the association between TBC1D10B expression and OS, PFI and DSS.

To verify the clinical benefits of TBC1D10B evaluation, we initially determined the predictive value on discriminating LIHC diagnosis by using an ROC curve. We observed that TBC1D10B showed significant sensitivity and specificity for LIHC prognosis (AUC = 0.931; Fig. 5A). Then, we performed *K-M* analyses to verify the predictions of TBC1D10B on clinical outcomes. As shown in Fig. 5B–D, LIHC patients with higher levels of TBC1D10B showed a significantly worse overall survival (HR = 1.59; *P* = 0.009), progression-free interval (HR = 1.36; *P* = 0.037), and disease-specific survival (HR = 1.78; *P* = 0.012).

**Figure 6:**
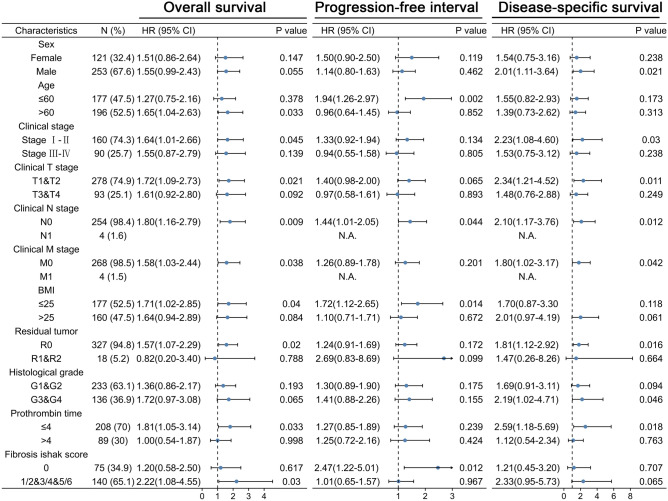
Prognostic performance of TBC1D10B on clinical outcomes in different LIHC patient subgroups. We divided patients into different subgroups based on the sex, age, clinical stage, clinical TNM stage, BMI, residual tumor, histological grade, prothrombin time, and fibrosis ishak score. For each subgroup, the prognostic performance of TBC1D10B on overall survival, disease-specific survival, and progression-free interval were evaluated by Cox regression, and the results are presented as hazard ratio. Error bar represents 95% confidence interval, and the size of diamond represents the significance of TBC1D10B’s performance.

Moreover, TBC1D10B was a significant risk factor for overall survival in several LIHC clinicopathological subgroups, including age above 60 years (HR = 1.65; *P* = 0.033), clinical stages I − II (HR = 1.64; *P* = 0.045), clinical stages T1 and T2 (HR = 1.72; *P* = 0.021), clinical N0 stage (HR = 1.80; *P* = 0.009), clinical M0 stage (HR = 1.58; *P* = 0.038), non-overweight (HR = 1.71; *P* = 0.04), no residual tumor (HR = 1.57; *P* = 0.02), prothrombin time less than 4 s (HR = 1.81; *P* = 0.033), and liver fibrosis (HR = 2.22; *P* = 0.03) (Table 3 and Fig. 6). Similar observations were seen for progression-free interval and disease-specific survival (Fig. 6).

**Table 3:** Prognostic performance of TBC1D10B on clinical outcomes in LIHC patient subgroup.

Characteristics	N (%)	HR for overall survival (95%Cl)	HR for progression-free interval (95%Cl)	HR for disease-specific survival (95%Cl)
**Sex**				
Female	121 (32.4)	1.51 (0.86–2.64)	1.50 (0.90–2.50)	1.54 (0.75–3.16)
Male	253 (67.6)	1.55 (0.99–2.43)	1.14 (0.80–1.63)	2.01 (1.11–3.64) *
**Age**				
≤60	177 (47.5)	1.27 (0.75–2.16)	1.94 (1.26–2.97) **	1.55 (0.82–2.93)
>60	196 (52.5)	1.65 (1.04–2.63)*	0.96 (0.64–1.45)	1.39 (0.73–2.62)
**Clinical stage**				
Stage I–II	160 (74.3)	1.64 (1.01–2.66)*	1.33 (0.92–1.94)	2.23 (1.08–4.60) *
Stage III–IV	90 (25.7)	1.55 (0.87–2.79)	0.94 (0.55–1.58)	1.53 (0.75–3.12)
**Clinical T stage**				
T1&T2	278 (74.9)	1.72 (1.09–2.73) *	1.40 (0.98–2.00)	2.34 (1.21–4.52) *
T3&T4	93 (25.1)	1.61 (0.92–2.80)	0.97 (0.58–1.61)	1.48 (0.76–2.88)
**Clinical N stage**				
N0	254 (98.4)	1.80 (1.16–2.79) **	1.44 (1.01–2.05) *	2.10 (1.17–3.76) *
N1	4 (1.6)	N.A.	N.A.	N.A.
**Clinical M stage**				
M0	268 (98.5)	1.58 (1.03–2.44) *	1.26 (0.89–1.78)	1.80 (1.02–3.17) *
M1	4 (1.5)	N.A.	N.A.	N.A.
**BMI**				
≤25	177 (52.5)	1.71 (1.02–2.85) *	1.72 (1.12–2.65) *	1.70 (0.87–3.30
>25	160 (47.5)	1.64 (0.94–2.89)	1.10 (0.71–1.71)	2.01 (0.97–4.19)
**Residual tumor**				
R0	327 (94.8)	1.57 (1.07–2.29) *	1.24 (0.91–1.69)	1.81 (1.12–2.92) *
R1&R2	18 (5.2)	0.82 (0.20–3.40)	2.69 (0.83–8.69)	1.47 (0.26–8.26)
**Histological grade**				
G1&G2	233 (63.1)	1.36 (0.86–2.17)	1.30 (0.89–1.90)	1.69 (0.91–3.11)
G3&G4	136 (36.9)	1.72 (0.97–3.08)	1.41 (0.88–2.26)	2.19 (1.02–4.71) *
**Prothrombin time**				
≤4	208 (70)	1.81 (1.05–3.14) *	1.27 (0.85–1.89)	2.59 (1.18–5.69) *
>4	89 (30)	1.00 (0.54–1.87)	1.25 (0.72–2.16)	1.12 (0.54–2.34)
**Fibrosis Ishak score**				
0	75 (34.9)	1.20 (0.58–2.50)	2.47 (1.22–5.01) *	1.21 (0.45–3.20)
1/2&3/4&5/6	140 (65.1)	2.22 (1.08–4.55) *	1.01 (0.65–1.57)	2.33 (0.95–5.73)

*HR* hazard ratio, *Cl* confidence interval.

*P < 0.05; **P < 0.01.

### Correlation between TBC1D10B expression, hypomethylation, and prognosis in LIHC

**Figure 7:**
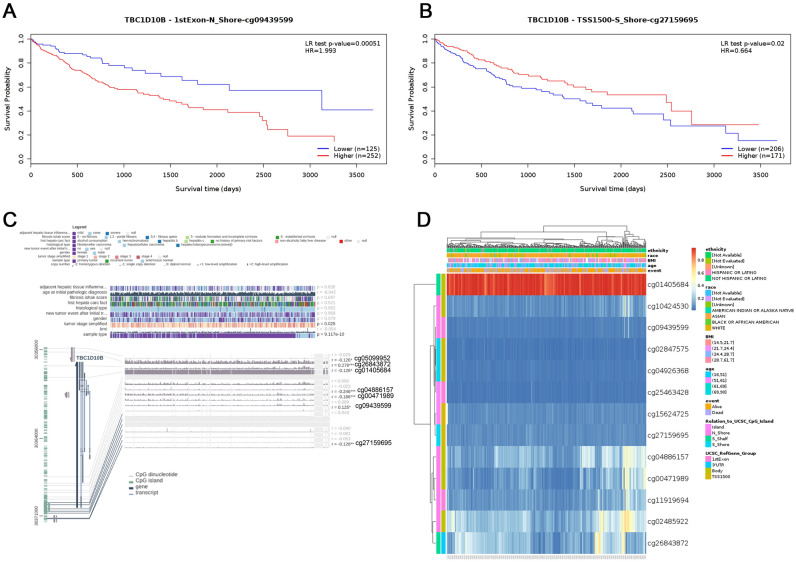
The MethSurv obtained the effect of hypomethylation level and TBC1D10B expression on prognosis in LIHC. (**A,B**) The Kaplan–Meier survival of the DNA methylation of TBC1D10B at cg09439599 and cg27159695 probes. (**C**) Association between SND1 DNA methylation and gene expression for the LIHC. (**D**) The visualization between the methylation level of TBC1D10B in LIHC.

We used the MEXPRESS approach to investigate the potential association between TBC1D10B DNA methylation and the pathogenesis of LIHC. As shown in Fig. 7C, we observed that TBC1D10B displayed a differential expression pattern in groups categorized according to simplified tumor stage and sample type. We also found a significant negative correlation of TBC1D10B expression and the methylation level of the cg09439599 (*P* < 0.05; R = 0.125) whereas it was positively correlated with the methylation level of the cg27159695 (*P* < 0.01; R = −0.128). Interestingly, the MethSurv analysis showed that patients with high methylation of cg09439599 had significantly worse survival outcomes than patients with low methylation (*P* < 0.001) while patients with high methylation of cg27159695 had a better survival outcome than patients with low methylation (*P* < 0.05) (Fig. 7A,B). Notably, the methylation levels of TBC1D10B are low in LIHC as shown by MethSurv (Fig. 7D). Taken together, these results suggest that alteration in DNA methylation levels may be the underlying mechanism responsible for the up- or downregulation of TBC1D10B in LIHC.

### Association of TBC1D10B and immune cell infiltration in LIHC tumor

**Figure 8:**
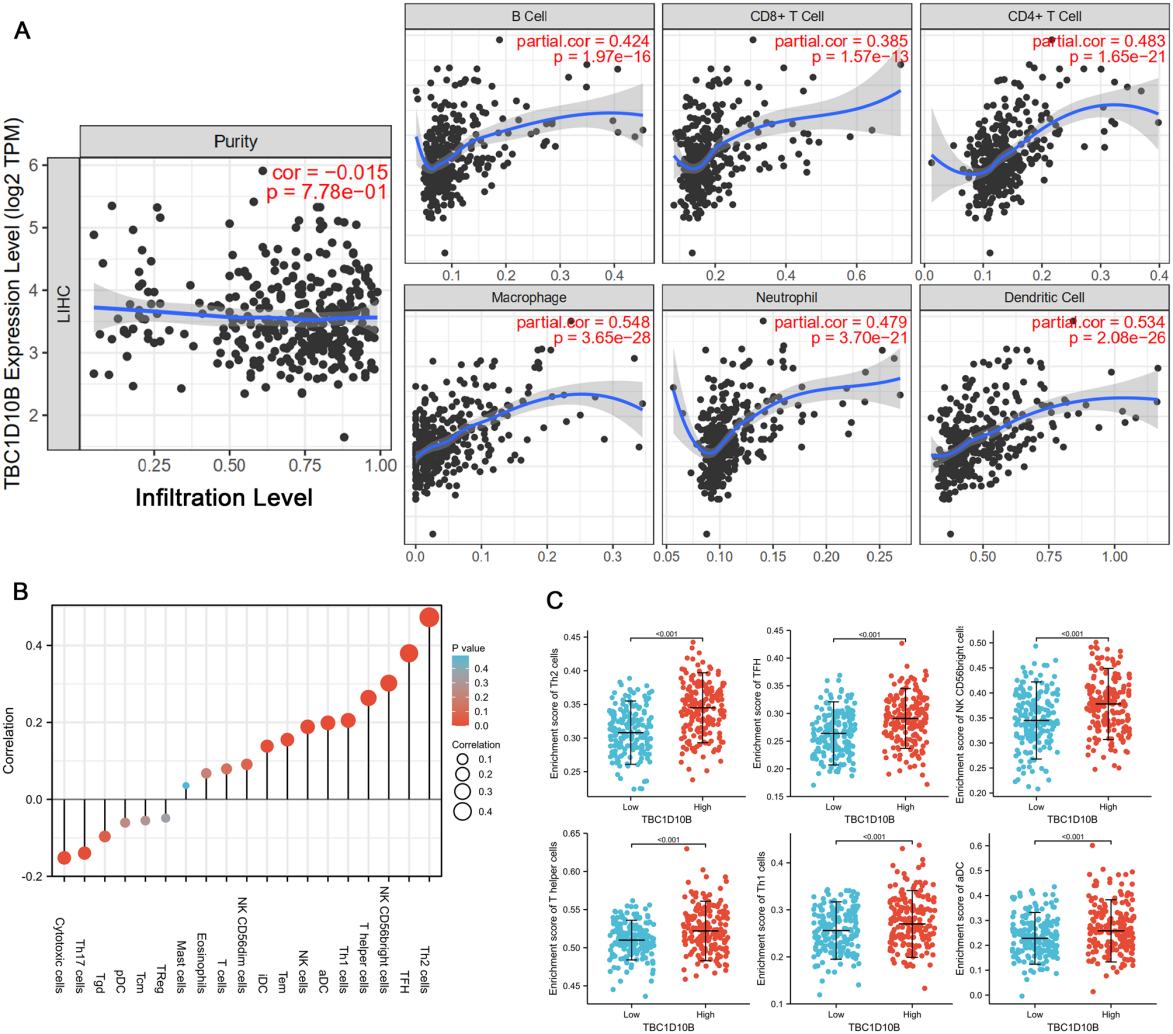
Figure 6. Correlation analysis between TBC1D10B expression and immune infiltration level. (**A**) TBC1D10B expression is positive related to B cell, CD8^+^ T cell, CD4^+^ T cell, neutrophil, macrophage, and dendritic cell (**A**). The correlation of TBC1D10B expression with the infiltration level of 19 immune cell types. Shown is the comparison of infiltration levels of most correlated immune cells, including type 2T helper (Th2) cells, T follicular helper (TFH) cells, natural killer (NK) CD56bright cells, T helper cells, Th1 cells, and aDC (**B,C**). *pDCs* plasmacytoid DCs, *iDCs* immature DCs, *aDCs* activated DCs, *Th* T helper cells, *Th17* type 17 Th cells, *Tgd* T gamma delta, *Tcm* T central memory, *Treg* regulatory T cells, *Tem* T effector memory, *NK* natural killer, *Th1* type 1 Th cells, *Th2* type 2 Th cells, *Tfh* T follicular helper.

As a prominent component of the tumor microenvironment, tumor-infiltrating immune cells are tightly correlated with the initiation, progression, and metastasis of cancer^35^. Herein, we observed a positive statistical correlation between TBC1D10B expression and the immune infiltration of B cells, CD4^+^ T cells, CD8^+^ cells, macrophages, neutrophils, and dendritic cells (Fig. 8A). Moreover, we determined the infiltration enrichment of other common immune cells by using the GSEA method from the R package “*GSVA*.” As shown in Fig. 8B, we observed a positive statistical correlation between TBC1D10B expression and the immune infiltrates of aDC (R = 0.205; *P* < 0.001), iDC (R = 0.0165; *P* = 0.001), NK CD56dim cells (R = 0.144; *P* = 0.005), NK cells (R = 0.270; *P* < 0.001), T helper cells (R = 0.278; *P* < 0.001), Tem (R = 0.202; *P* < 0.001), Tfh (R = 0.0391; *P* < 0.001), Th1 (R = 0.241; *P* < 0.001), and Th2 (R = 0.470; *P* < 0.001), but cytotoxic cells (R = −0.153; *P* = 0.003) and Th17 (R = −0.247; *P* < 0.001) exhibited a significantly negative TBC1D10B expression (Fig. 8C).

## Discussion

TBC1D10B is a ubiquitously expressed member of the EPI64 subfamily responsible for inactivating Ras and certain Rabs, but studies on TBC1D10B have focused mainly on its GAP activities, such as Rab3A, Rab22, Rab35, and Rab22^23–25^. Previous studies have shown that TBC1D10B serves as an essential downstream target gene of miR-3619-5p and regulates stomach adenocarcinoma progression^28^, indicating an intimate link between aberrant TBC1D10B expression and cancer development and progression. Unfortunately, whether dysregulation of TBC1D10B occurs following cancer occurrence has not been fully revealed. In our study, the TBC1D10B expression in most common cancers, including breast cancer and lung adenocarcinoma, was significantly elevated.

Similar to other GAPs^36^, TBC1D10B has been implicated in cancers^17,37^. Our study has, for the first time, shown that TBC1D10B is overexpressed at mRNA levels in LIHC. We then found that the primary tumors had an increased level of phosphorylated TBC1D10B at the S132 site compared with normal controls but not an increased total TBC1D10B protein level. Moreover, we verified the mRNA and protein levels of TBC1D10B in cells. The discordance between protein and mRNA levels indicates that the elevated RNA levels of TBC1D10B may be common, but they may not correlate with actual total protein expression or responses to certain cancer types. Recently, Cartery et al. reported phosphoprotein-based biomarkers as significant predictors for cancer therapy^38^; we asked whether the phosphorylation levels of TBC1D10B might be useful for predicting the prognosis of LIHC patients. We cannot, of course, rule out the possibility that the high TBC1D10B phosphorylation level of S132 is a byproduct of dysregulated signaling without functional significance in LIHC. We also identify DEGs including mRNAs, miRNAs, and lncRNAs, between the low and high TBC1D10B expression groups. Considering that noncoding RNAs have been identified as oncogenic drivers and tumor suppressors in every major cancer type^39–41^, we postulated that TBC1D10B may interact with some miRNAs or lncRNAs, thus controlling LIHC carcinogenesis and progression, although further investigations are needed.

Increased cell proliferation and loss of cell adhesion are two common features of malignant cancer cells^42,43^. The alterations in both the density and composition of the ECM are reported as potential targets for resistant tumors, leading to tumor heterogeneity and enhancing tumor recurrence^44^. TBC1D10B has been indicated to serve as RabGAP for Rab22a^23^, which mediate formation of extracellular vesicles that contribute to tumor microenvironment remodeling^45^ and can promote tumor progression^46^. In this paper, our results show that the DEGs related to higher TBC1D10B levels are enriched specifically in the cell cycle, MET-associated pathway, and ECM regulation, suggesting that an alteration in TBC1D10B expression may be a cause of changing both cancer cells’ phenotypes and the tumor microenvironment.

Similar to a previous report in which high TBC1D10B expression was linked to a bad prognosis in stomach adenocarcinoma patients, we reveal a significant correlation of TBC1D10B expression with gender, BMI, histologic grade, T stage, pathological stage, AFP, tumor status, and residual tumor in LIHC patients, supporting the pro-oncogenic characteristics of TBC1D10B in liver cancer development. However, it should be noted that TBC1D10B protein expression was upregulated by the traditional Chinese medicine tongxinluo, which has a protective role in response to ischemia/reperfusion injury^47^. The finding that TBC1D10B can play both a positive and a negative role in a pathological condition seems contradictory. Nevertheless, previous studies have demonstrated that Rab27B can be inactivated by high TBC1D10B expression^48^. Moreover, a decreased expression of Rab27B could play an oncogenic role in colorectal cancer^49^. Thus, we postulate that an oncogenic effect could be triggered in an indirect manner by TBC1D10B and that it may have a direct protective effect in heart repair after injury via the TBC1D10B-binding mechanism.

Despite the rapid growth in therapeutic and diagnostic technology^50^, the improvement of liver cancer prognosis is still disappointing^2^. In our study, the ROC curve for TBC1D10B discrimination of LIHC diagnosis had an AUC of 0.931. Meanwhile, our study for the first time demonstrated that high TBC1D10B expression is related to poor overall survival, progression-free interval, and disease-specific survival. These findings strongly indicate that TBC1D10B may act as a convincing biomarker for LIHC diagnosis. In addition, the prognostic value of TBC1D10B seemed to be more prominent in LIHC patients with certain features, including weight below 70 kg, height below 170 cm, and histological G2 − G3 stage (Supplemental Fig. 3). Our findings may provide possible research directions for prognostic assessment in LIHC.

DNA methylation reportedly regulates cell differentiation and participates in tumorigenesis^51^. Based on genome-wide methylation profiling, Villanueva et al.^52^ found that a 36-probe methylation signature can accurately predict survival in LIHC patients. Herein, we found a low TBC1D10B methylation and a potential correlation between high TBC1D10B expression and low DNA methylation status in LIHC patients. Interestingly, we observed that TBC1D10B methylation at the promoter region (cg27159695) was associated with a poor prognosis of LIHC while TBC1D10B methylation at the non-promoter region (cg09439599) correlated with a good prognosis. Therefore, additional evidence is needed of the potential role of TBC1D10B DNA methylation in the tumorigenesis of LIHC.

Because LIHC is an inflammation-associated tumor, the immunosuppressive microenvironment of LIHC contributes to immune escape and tolerance through multiple routes. Substantial clinical data and experimental research have confirmed that tumor initiation, tumor progression, and malignant metastasis are related to macrophages^53^ and neutrophils^54^. As dedicated professional antigen-presenting cells, dendritic cells can prime T-cells against tumor-associated antigens, which are involved in the progression of hepatocellular carcinoma^55^. Moreover, an increased number of both circulating and intra-tumoral CD8^+^ T cells in hepatocellular carcinoma predicts high postoperative recurrences and poor prognoses^56^. Our study has for the first time demonstrated that high TBC1D10B expression is positively correlated with CD8^+^ T cells, B cells, CD4^+^ T cells, macrophages, neutrophils, dendritic cells, and most other immune cells. These findings indicate that the dysfunction of TBC1D10B may contribute to cancer cell immune escape. Notably, we found a significantly negative correlation between TBC1D10B expression and cytotoxic cell infiltration, which are the preferred immune cells for targeting cancer^57^. Therefore, the roles and functions of TBC1D10B need to be further studied to provide a new strategy for tumor immunotherapy.

Our combined findings show for the first time that the mRNA expression of TBC1D10B is upregulated in LIHC and significantly associated with clinical prognosis, DNA methylation, and immune cell infiltration. In addition, we uncovered a potential mechanism for TBC1D10B activity in LIHC tumorigenesis and demonstrated its predictive value in LIHC clinical outcomes. Our research suggests that TBC1D10B may be regarded as a novel prognostic and predictive marker and therapeutic target for hepatocellular carcinoma patients, including RNA-targeting treatment, such as antisense oligonucleotides or RNA-targeting Cas9.

## Supplementary Information

Supplementary Information 1.

Supplementary Information 2.

Supplementary Information 3.

Supplementary Information 4.

Supplementary Figures.

Supplementary Information 5.

Supplementary Information 6.

## Data Availability

The raw data supporting the conclusion of this article will be made available by the corresponding author (a17377552242@163.com), without undue reservation.
