# Supplementary material for: Increased expression of TBC1D10B as a potential prognostic and immunotherapy relevant biomarker in liver hepatocellular carcinoma
Source: Sci Rep. 2023 Jan 7;13:335. doi: 10.1038/s41598-022-20341-1 (PMC9825366; doi:10.1038/s41598-022-20341-1)
Supplement: Supplementary file 5 — Supplementary Figures. [file 41598_2022_20341_MOESM5_ESM.docx]

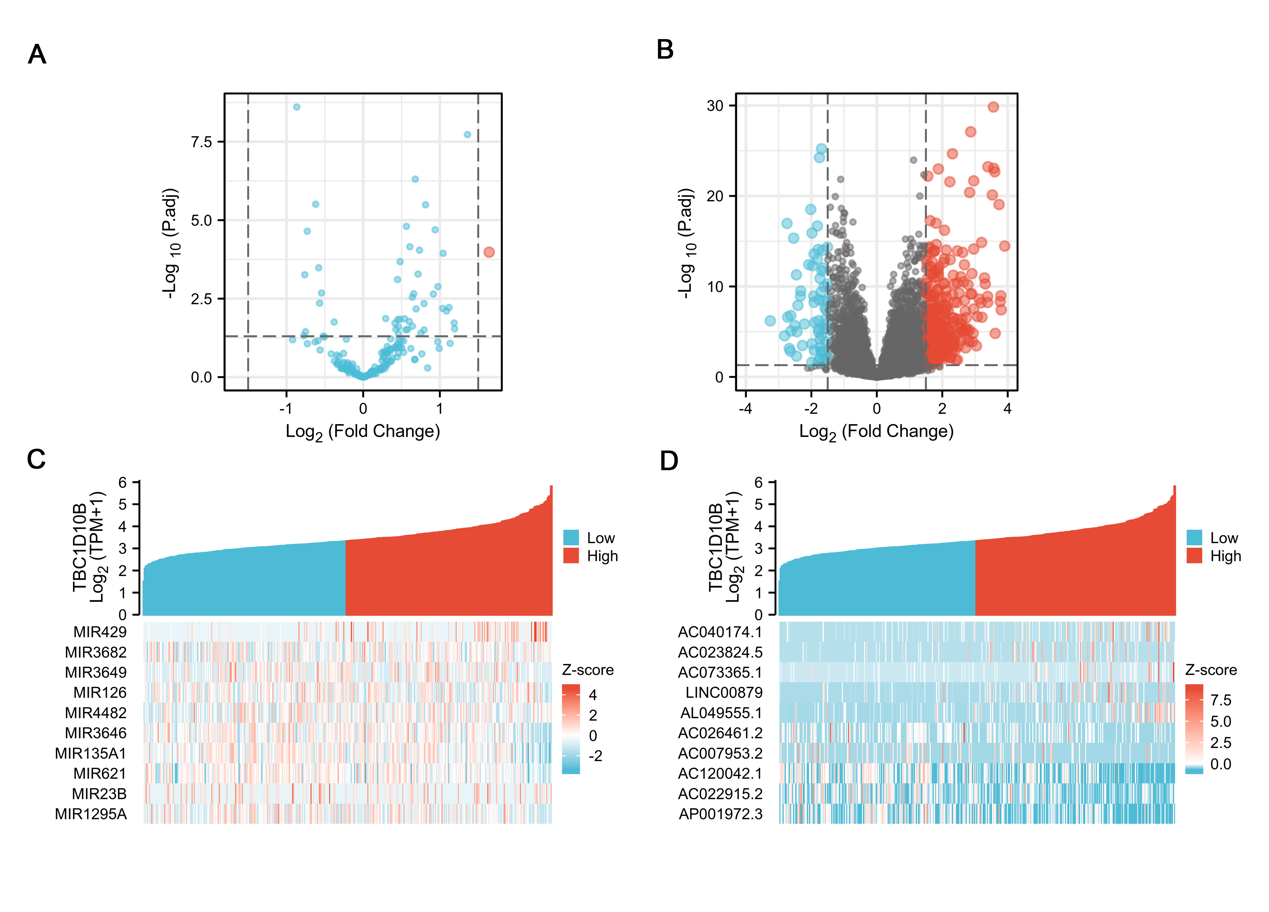


**Supplementary Figure 1:** Differential miRNA and lncRNAs expression profiles in liver hepatocellular carcinoma (LIHC) patients. (**A-B**) A heat map (**C-D**) depicting the miRNA and lncRNAs expression profiles are shown. Differentially expressed genes that are up- or downregulated at least 1.5 fold.


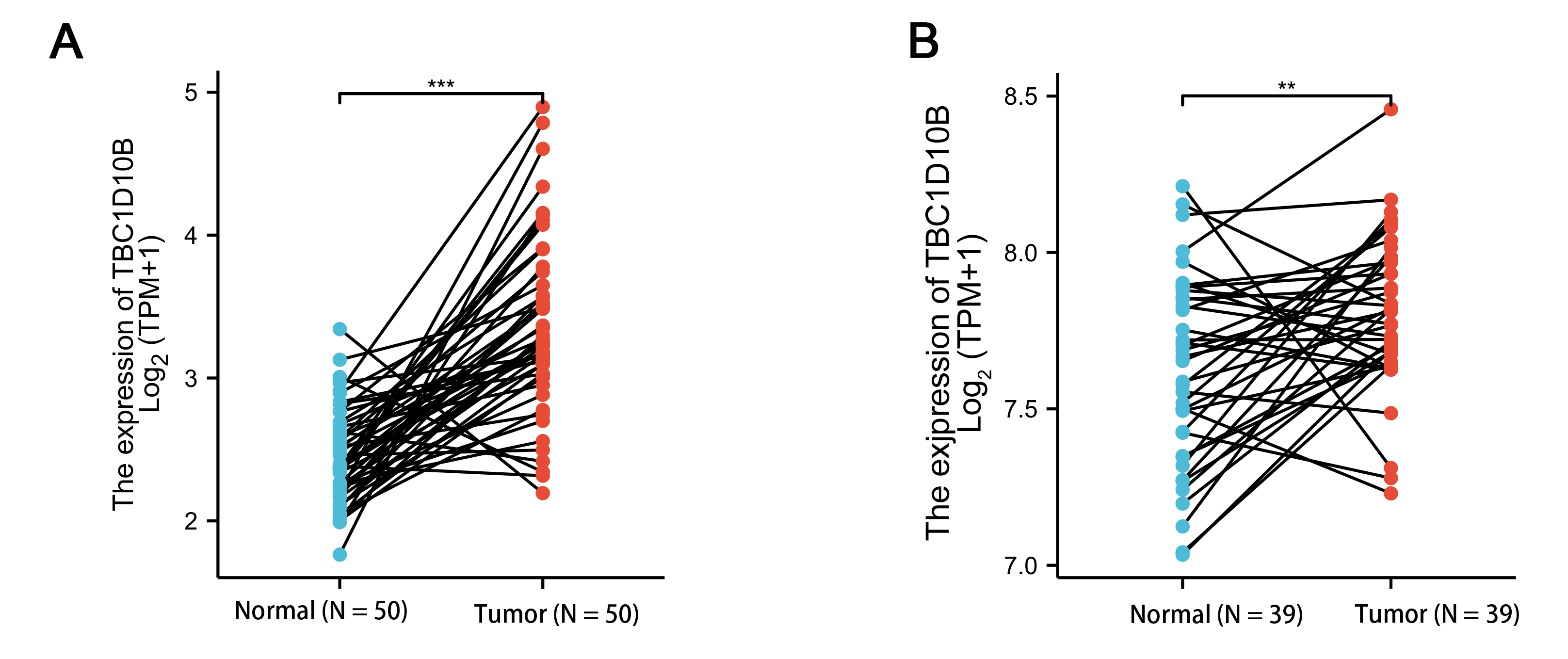


**Supplementary Figure 2: (A-B) Expression of TBC1D10B mRNA in LIHC and matched normal samples in TCGA and GEO databases.**


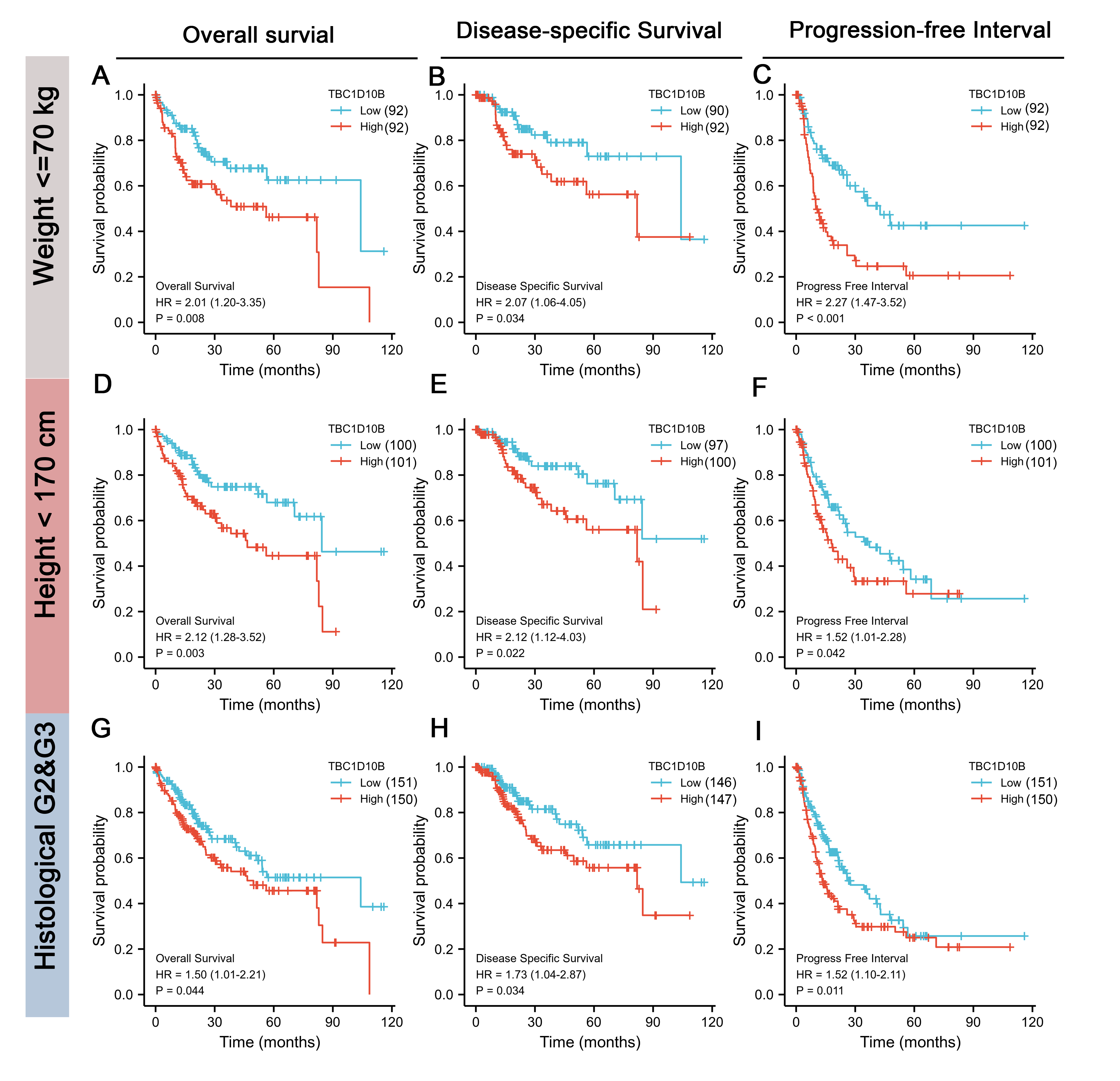


**Supplementary Figure 3:** Distinct clinical outcomes based on TBC1D10B expression in liver hepatocellular carcinoma (LIHC) patients. Kaplan-Meier analysis showing the comparison of overall survival (**A, D, G**), disease-specific survival (**B, E, H**), and progression-free interval (**C, F, I**) between high- and low-TBC1D10B expression groups in several LIHC patient subgroups, including weight less than or equal to 70 kg (**A-C**), height less than or equal to 170 cm (**D-F**), and histological G2-G3 (**G-I**). *P* < 0.05 was considered statistically significant.
