# Supplementary figures and images for: Increased expression of TBC1D10B as a potential prognostic and immunotherapy relevant biomarker in liver hepatocellular carcinoma
Source: Sci Rep. 2023 Jan 7;13:335. doi: 10.1038/s41598-022-20341-1 (PMC9825366; doi:10.1038/s41598-022-20341-1)

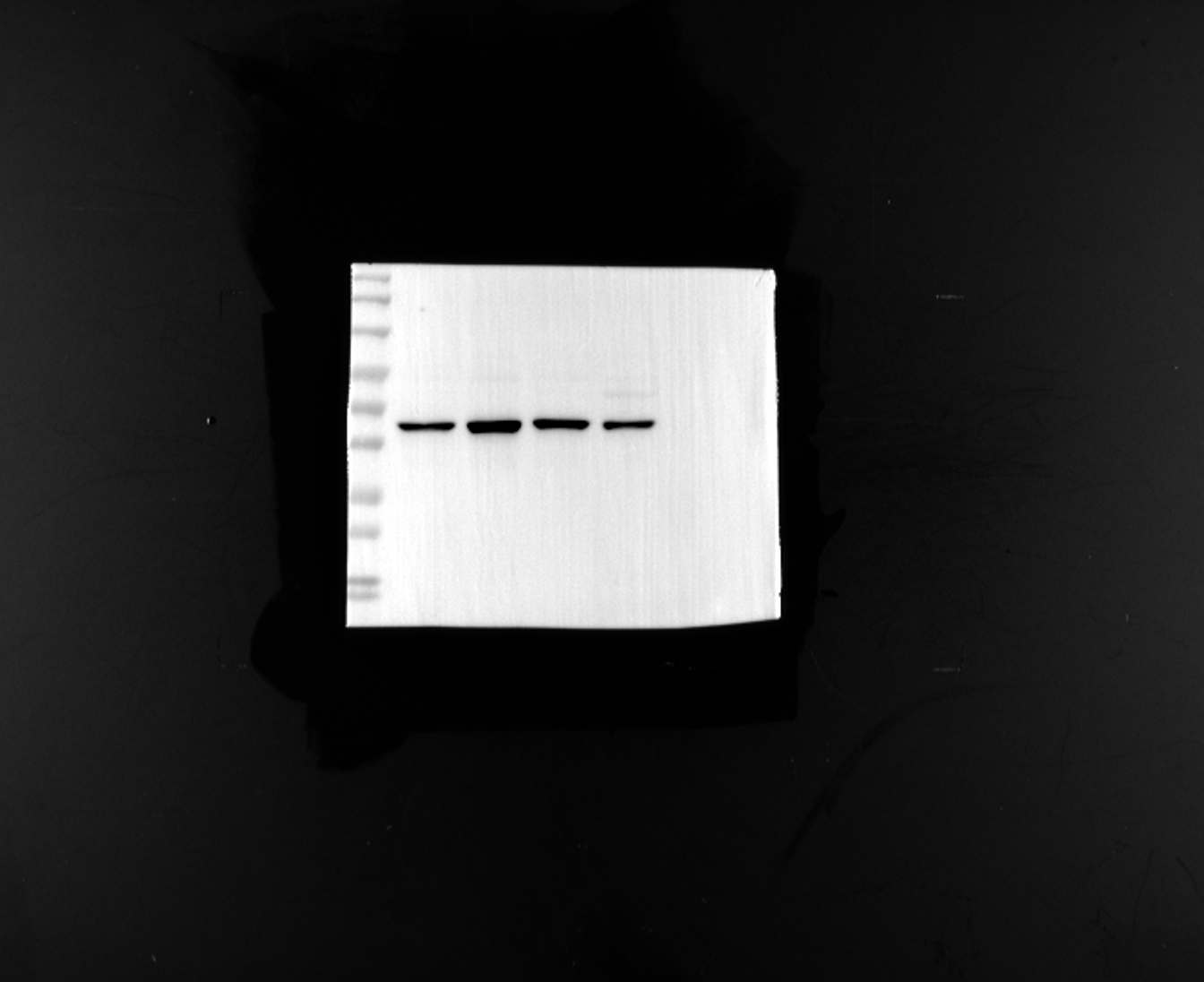

Supplement: Supplementary file 6 — Supplementary Information 5. [file 41598_2022_20341_MOESM6_ESM.tif]

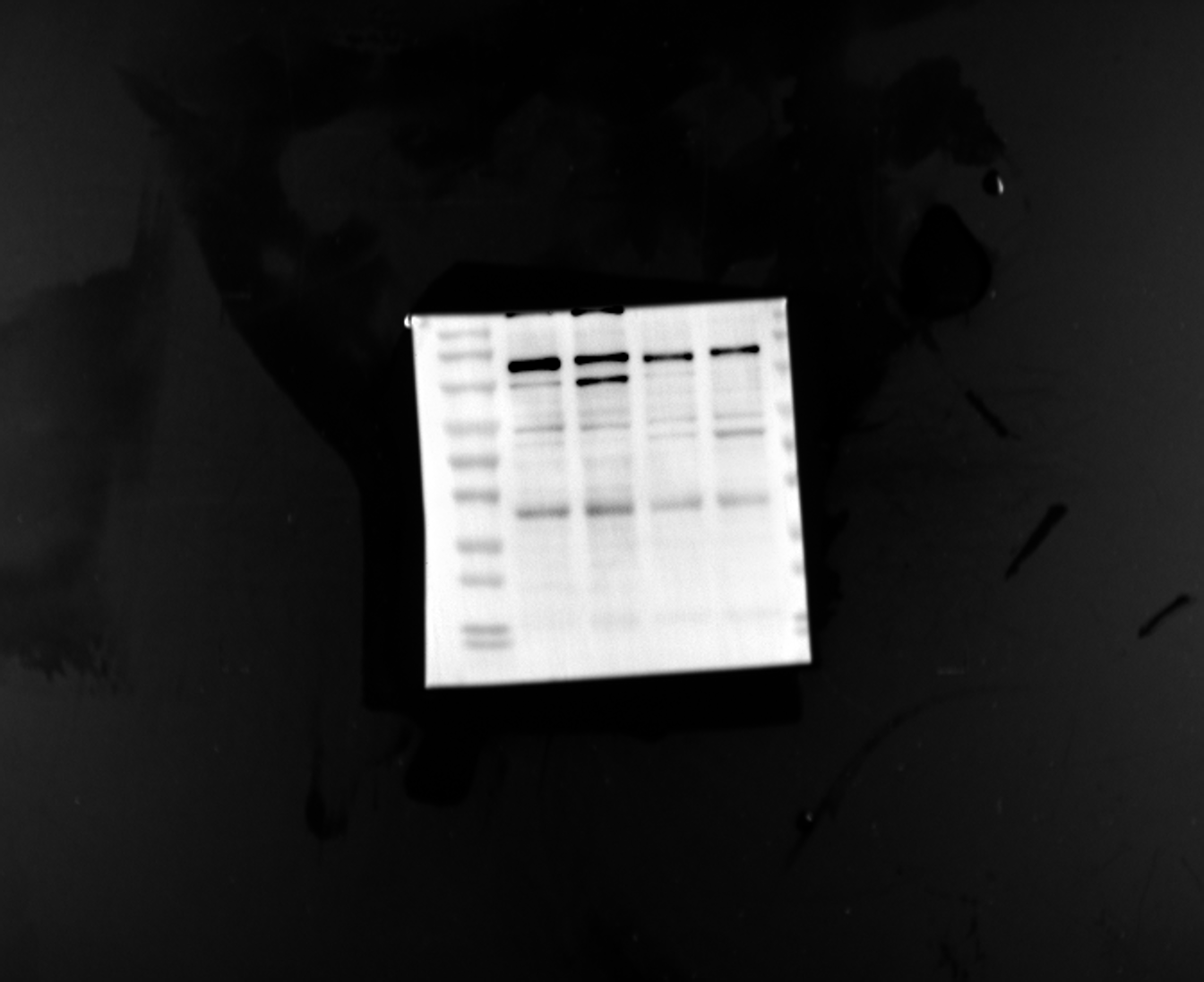

Supplement: Supplementary file 7 — Supplementary Information 6. [file 41598_2022_20341_MOESM7_ESM.tif]
